# *In Utero* Exposure to Low-Dose Alcohol Induces Reprogramming of Mammary Development and Tumor Risk in MMTV-erbB-2 Transgenic Mice

**DOI:** 10.3390/ijms16047655

**Published:** 2015-04-07

**Authors:** Zhikun Ma, Amanda J. Blackwelder, Harry Lee, Ming Zhao, Xiaohe Yang

**Affiliations:** Julius L. Chambers Biomedical/Biotechnology Research Institute and Department of Biology, North Carolina Central University, Kannapolis, NC 28081, USA; E-Mails: mzhao@nccu.edu (Z.M.); ablack18@nccu.edu (A.J.B.); hlee11@nccu.edu (H.L.); mazhikun1225@163.com (M.Z.)

**Keywords:** alcohol, *in utero* exposure, breast cancer risk, estrogen receptor, erbB-2, reprogramming

## Abstract

There is increasing evidence that prenatal exposure to environmental factors may modify breast cancer risk later in life. This study aimed to investigate the effects of *in utero* exposure to low-dose alcohol on mammary development and tumor risk. Pregnant MMTV-erbB-2 mice were exposed to alcohol (6 g/kg/day) between day 13 and day 19 of gestation, and the female offspring were examined for tumor risk. Whole mount analysis indicated that *in utero* exposure to low-dose alcohol induced significant increases in ductal extension at 10 weeks of age. Molecular analysis showed that *in utero* alcohol exposure induced upregulation of ERα signaling and activation of Akt and Erk1/2 in pubertal mammary glands. However, enhanced signaling in the EGFR/erbB-2 pathway appeared to be more prominent in 10-week-old glands than did signaling in the other pathways. Interestingly, tumor development in mice with *in utero* exposure to low-dose alcohol was slightly delayed compared to control mice, but tumor multiplicity was increased. The results indicate that *in utero* exposure to low-dose alcohol induces the reprogramming of mammary development by mechanisms that include altered signaling in the estrogen receptor (ER) and erbB-2 pathways. The intriguing tumor development pattern might be related to alcohol dose and exposure conditions, and warrants further investigation.

## 1. Introduction

The *in utero* stage is a vulnerable window in which organisms are very sensitive to environmental factors [1]. Increasing evidence indicates that maternal exposure to various dietary and environmental factors during pregnancy has a profound impact on the offspring’s mammary development and breast cancer risk [2]. A notable example is the DES (diethylstilbestrol) daughter story; the daughters of women exposed to DES during pregnancy have an increased risk of vaginal adenocarcinoma and breast cancer [3]. Animal studies have shown that *in utero* exposure to a number of factors, such as bisphenol A (BPA) and *n*-6 polyunsaturated fatty acids (PUFA), increase mammary tumor risk [4,5]. In contrast, maternal exposure to soy protein isolate and *n*-3 PUFA was associated with decreased mammary tumor risk in the DMBA carcinogen model [6,7]. These studies underscore the significance of prenatal factors in breast cancer risk later in life.

Although alcohol intake has been associated with increased breast cancer risk, most studies were based on the risk of the individuals who consumed the alcohol [8,9]. Studies on the impact of mothers’ alcohol consumption during pregnancy on daughters’ mammary development and breast cancer risk, however, remain sparse. Nevertheless, the CDC reported that 7.6% of pregnant women admitted drinking alcohol during pregnancy [10]. *In utero* exposure to alcohol has been associated with developmental damage or diseases such as fetal alcohol syndrome (FAS) [11]. With increasing reports on the modification of tumor risk later in life by *in utero* exposure, it is imperative to understand whether and how *in utero* exposure to alcohol modifies breast cancer risk. Hilakivi-Clark *et al.* first reported that *in utero* exposure to alcohol via liquid diets containing 16–25 g alcohol per kg fed between days 7 and 19 of gestation promoted 7,12-dimethylbenz(a)anthracene (DMBA)-induced mammary tumor development in Sprague-Dawley rats [12]. Polanco *et al.* found that *in utero* alcohol exposure via liquid diets containing 6.7% (*v*/*v*) alcohol increased mammary tumor susceptibility to *N*-nitroso-*N*-methylurea (NMU)-mediated carcinogenesis in rats, as indicated by greater multiplicity and more malignant phenotypes [13]. The results from the above studies indicated that the modified mammary tumor risk associated with *in utero* exposure to alcohol was associated with increased serum estradiol levels in alcohol-exposed mothers, as well as enhanced prepubertal mammary development and deregulated IGF-I and estradiol systems in their offspring [12,13,14]. These changes appear to be similar to the alterations induced by *in utero* exposure to hormonal disruptors [12].

Although earlier studies provided “proof of concept” for that *in utero* exposure to alcohol could modify mammary tumor risk, the molecular mechanisms by which *in utero* exposure to alcohol modifies mammary development and tumor risk remain largely unknown. Factors that may affect the effects of *in utero* alcohol exposure, such as genetic predisposition, have not been documented. To facilitate mechanistic studies, a transgenic model with a defined genetic background and clinically relevant predisposition may provide further insight into the underlying mechanisms. We therefore tested the effect of *in utero* exposure to low-dose alcohol on mammary tumor risk in MMTV-erbB-2 transgenic mice.

erbB-2 (Her2/neu) is a member of the ErbB family of receptor tyrosine kinases (RTKs), which also includes EGFR, erbB-3 and erbB-4 [15]. Aberrant expression/activation of these RTKs plays a critical role in breast cancer development. In particular, erbB-2 is amplified/overexpressed in approximately 30% of human breast cancers, and this effect has been associated with poor prognosis and therapeutic resistance [16,17]. Activated erbB-2 interacts with its family members to induce the activation of a plethora of pathways, such as the PI3K/Akt and MAPK/Erk pathways, that are involved in cell proliferation, survival and other activities [18,19,20]. The MMTV-erbB-2 transgenic mouse is a well-established model for studies on the effect of various environmental, dietary and genetic factors on erbB-2-mediated carcinogenesis [21,22]. Using this model, we demonstrated an interaction between estrogen and erbB-2 induced mammary tumor development in transgenic mice [23]. We also showed that low doses of soy isoflavones interfered with tamoxifen-mediated chemoprevention in MMTV-erbB-2 mice [24], indicating that this model can detect subtle oncogenic factors. Moreover, Wong *et al.* reported that postnatal alcohol consumption promoted mammary tumor development in MMTV-erbB-2 transgenic mice through the estrogen pathway [25]. These studies suggest that MMTV-erbB-2 transgenic mice could be a useful model for studying the modification of mammary development and tumor risk by *in utero* exposure to alcohol.

We describe here, for the first time, the use of MMTV-erbB-2 transgenic mice to examine the effects of *in utero* alcohol exposure on mammary development and tumor risk. We found that *in utero* exposure to low-dose alcohol induced prolonged ductal extension and altered differentiation of mammary glands. Although the treatment increased tumor multiplicity, tumor development was initially delayed under the given conditions. Molecular analysis indicated that *in utero* alcohol exposure induced the upregulation of the ER and the erbB-2 pathways in mammary tissues. These results also provide support for using the MMTV-erbB-2 transgenic mouse model for studies on *in utero* exposure to alcohol-modified mammary tumor development.

## 2. Results

### 2.1. Effect of Alcohol Exposure on Plasma Alcohol and E2 Levels in Pregnant Mice

**Figure 1:**
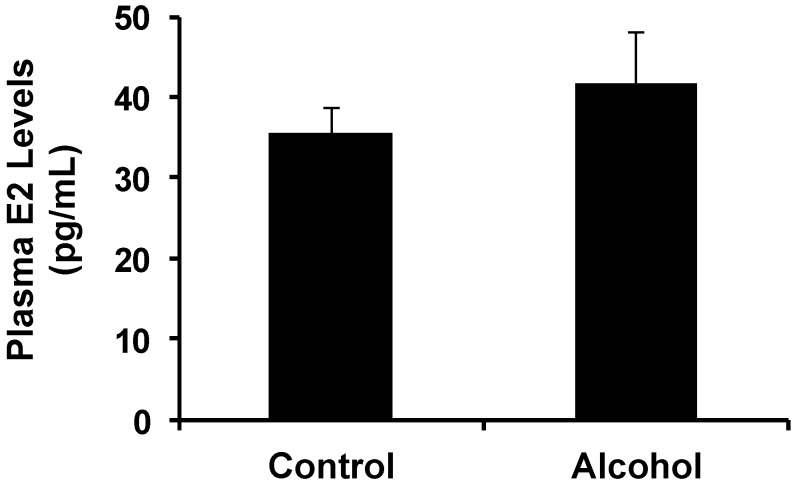
Plasma E2 levels on day 18 of gestation in MMTV-erbB-2 mice with alcohol exposure. The mice were exposed to alcohol via oral feeding of 0.5 mL of 10% (days 10–12) or 20% (days 13–19) alcohol/water twice a day. *n* = 5 animals per group. *p* = 0.09.

To examine the effect of alcohol exposure on plasma alcohol and E2 levels, pregnant mice in the control and alcohol groups were exposed to water and alcohol as detailed in the methods; blood samples were collected from each group on day 18 of gestation. Thirty minutes after alcohol administration, the blood alcohol concentration (BAC) of the mice in the alcohol group was 114.5 ± 12.6 mg/dL, which was similar to the BAC of mice given 1.12 g/kg alcohol [26]. Moreover, the determination of plasma E2 levels indicated that *in utero* exposure to alcohol under the given conditions induced a modest increase in circulating E2 levels (Figure 1), although it was not significant, as it was in a report with high dose exposure [12].

### 2.2. Effect of in Utero Exposure to Low-Dose Alcohol on Body Weight and Vaginal Opening Date

**Figure 2:**
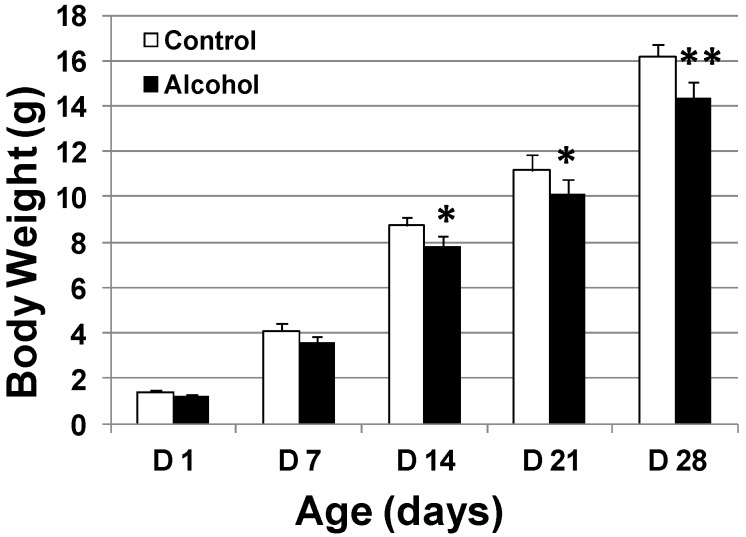
*In utero* exposure to alcohol results in lower body weight. MMTV-erbB-2 mice were exposed to 3 g/kg/day alcohol between day 10 and day 12 and 6 g/kg/day alcohol between day 13 and day 19 of gestation by feeding. The body weight of the pups was measured weekly for the first 4 weeks. * *p* < 0.05; ** *p* < 0.01.

Prenatal alcohol exposure may induce both local and systemic changes. We therefore examined the body weight and vaginal opening date of newborns that had been exposed to different *in utero* treatments. Weekly measurement of the newborns’ body weight indicated that the pups that had been exposed to alcohol *in utero* had a lower body weight than the control pups (Figure 2), suggesting that *in utero* alcohol exposure under the given conditions may affect general development. 

Vaginal opening is an indicator of puberty onset. Earlier puberty onset has been observed in animals after *in utero* exposure to hormonal disruptors [27]. We examined the effect of *in utero* exposure to low-dose alcohol on vaginal opening in the MMTV-erbB-2 transgenic mice. As shown in Table 1, the difference in vaginal opening dates between the two groups was not significant.

**Table 1:** Effect of *in utero* exposure to alcohol on date of vaginal opening.

Group	*n*	D27	D28	D29	D30	D31	Average *
Control	20	0	4	6	8	2	29.40 ± 0.94
Alcohol	22	2	6	6	8	0	28.90 ± 1.01

Vaginal opening of control and alcohol-exposed mice was examined daily between day 22 and day 33 after birth. * Average date of vaginal opening.

### 2.3. In Utero Exposure to Low-Dose Alcohol Induces Modified Mammary Morphogenesis in MMTV-erbB-2 Mice

**Figure 3:**
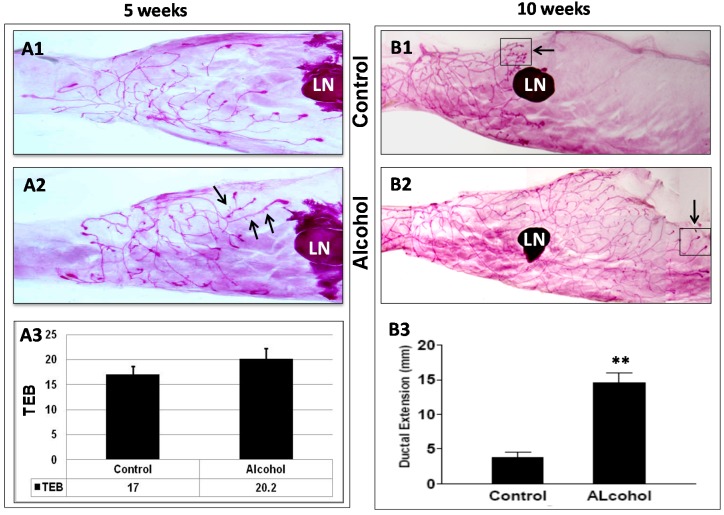
*In utero* exposure to alcohol induces prolonged mammary ductal extension. Mammary whole mounts were harvested from control and alcohol-exposed mice at 5 and 10 weeks of age and stained with carmine red (**A1**,**A2**,**B1**,**B2**). The number of terminal end buds (TEBs) at 5 weeks (**A3**) and the ductal extension beyond the lymph node (LN) at 10 weeks (**B3**) were quantified. Six glands from each group were examined. ** *p* < 0.01.

*In utero* exposure to higher doses of alcohol has been reported to increase the number of terminal end buds (TEBs) and mammary epithelial densities in rats, as evaluated with a multiparameter system [12,28]. We analyzed the mammary morphology of 5- and 10-week-old erbB-2 transgenic mice from our two groups. At 5 weeks of age, mammary glands in mice that had been exposed to alcohol *in utero* appeared to display more advanced development when compared to those of control mice (Figure 3A1), as indicated by more lateral buds along the mammary ducts, though this parameter was not quantified (arrows in Figure 3A2). The difference of the TEB numbers between the two groups, however, was not significant (Figure 3A3).

At 10 weeks of age, the mammary morphology of mice that had been exposed to alcohol *in utero* showed striking differences from that of the control mice. In the control MMTV-erbB-2 mice, mammary ductal tree elongation at 10 weeks was relatively inhibited compared with parental FVB mice at the same age (Yang, X., unpublished data). After *in utero* exposure to low-dose alcohol, however, the most prominent change in these mice was prolonged ductal tree extension in the mammary fat pad (Figure 3B2). Using the lymph node (LN) as a landmark, we determined that mammary ductal extension beyond the lymph node of the glands in the alcohol-exposed group was significantly greater than that of the controls (Figure 3B3). Another notable feature was that the number of lateral buds and alveolar structures in the distal end of the ductal tree was smaller in the alcohol-exposed group than in the control group. In particular, many of the TEBs in the glands of the alcohol group resembled TEBs in pubertal glands, as indicated by the presence of enlarged ends and small numbers of lateral buds (squares and arrows in Figure 3B1,B2). The results suggest that *in utero* exposure to low-dose alcohol induces not only ductal elongation but also modified differentiation, and provide morphologic evidence of reprogramming induced by *in utero* alcohol exposure.

### 2.4. In Utero Exposure to Alcohol Induces ER Pathway Signaling in Pubertal Mammary Glands

**Figure 4:**
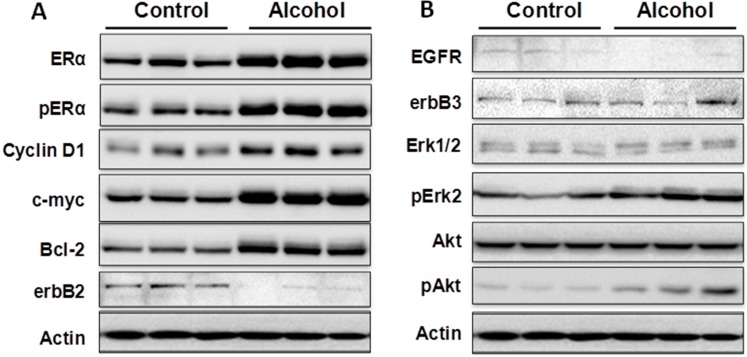
*In utero* exposure to low-dose alcohol induces activation of the ER pathway in pubertal mammary glands. Protein lysates were prepared from mammary tissue from five-week-old mice after *in utero* treatments. Protein levels of the indicated markers of the ER and erbB-2 pathways were detected using Western blotting (**A**,**B**). Each group contained three samples from different mice that had undergone the same treatment.

To understand the molecular mechanisms associated with alterations in mammary morphogenesis induced by *in utero* exposure to alcohol, we analyzed molecular signaling in the estrogen receptor (ER) and erbB-2 pathways of mammary tissue from mice exposed to different treatments. Examination of markers of the ER pathway in pubertal mammary tissues (5 weeks of age) showed that the protein levels of both ERα and phosphorylated-ERα (p-ERα) were increased in the alcohol group (Figure 4). Concurrently, protein levels of ER transcriptional targets, including cyclin D1, c-myc and Bcl-2, were also significantly increased. These results suggest that ERα expression and activation in pubertal mammary glands are sensitive targets of molecular changes induced by *in utero* alcohol exposure.

Analysis of key markers associated with erbB-2 signaling indicated that *in utero* alcohol exposure induced a modest decrease in erbB-2 and EGFR in the pubertal glands (Figure 4). Nevertheless, the levels of phosphorylated Akt and Erk (pAkt and pErk), which are downstream of the receptor tyrosine kinase cascade, were increased in the alcohol group, despite the mixed expression profile at the receptor level. The data suggest that *in utero* exposure to alcohol may modulate erbB-2 signaling at multiple levels. The increased pAkt and pErk levels suggest that *in utero* alcohol exposure under the given conditions results in a modest overall downstream activation.

### 2.5. In Utero Exposure to Alcohol Induces the Transcription of ER Target Genes and EGFR Ligands in Pubertal Mammary Glands

**Figure 5:**
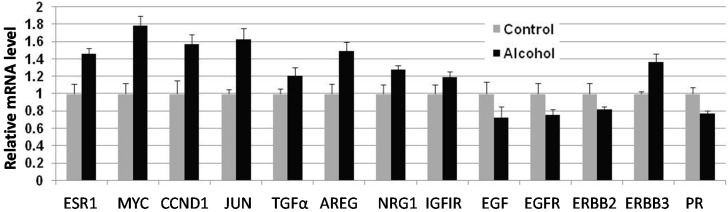
*In utero* exposure to alcohol induced transcriptional regulation of ER target genes and growth factor receptors and cognate ligands. Total RNA was extracted from mammary tissue from five-week-old MMTV-erbB-2 mice after different *in utero* treatments. Equal amounts of RNA from the third pair of glands from four different mice were pooled for further analysis. The relative mRNA levels of individual genes were detected by qRT-PCR in triplicate. Bars represent standard error of triplicate measurements in the qRT-PCR assay.

To examine molecular changes induced by *in utero* alcohol exposure at the transcriptional level, we measured the mRNA levels of a panel of genes involved in the ER, progesterone receptor (PR) and growth factor pathways in mammary tissue from five-week-old mice (Figure 5). Among these genes, ERα/*ESR1*, c-myc/*MYC*, cyclinD1/*CCND1* and *c-Jun* are typical ER target genes. EGFR, erbB-2 (endogenous) erbB-3, TGFα, amphiregulin (Areg) and neuregulin 1 (NRG1) are all either erbB family receptors or cognate ligands. The results showed that the transcription of ESR1/ERα, c-myc, cyclinD1/CNND1, c-Jun, TGFα, Areg, NRG1 and erbB-3 in the alcohol group was upregulated. However, the transcription of PR, EGF, EGFR and erbB-2 was downregulated. Taken together, these changes suggest that *in utero* exposure to low-dose alcohol induces consistent activation of the ER pathway and a mixed profile of activation of the erbB-2 pathway in mammary tissues at puberty.

### 2.6. In Utero Alcohol Exposure Induces Modified Signaling in Growth Factor Pathways in Mammary Tissues of 10-Week-Old Mice

**Figure 6:**
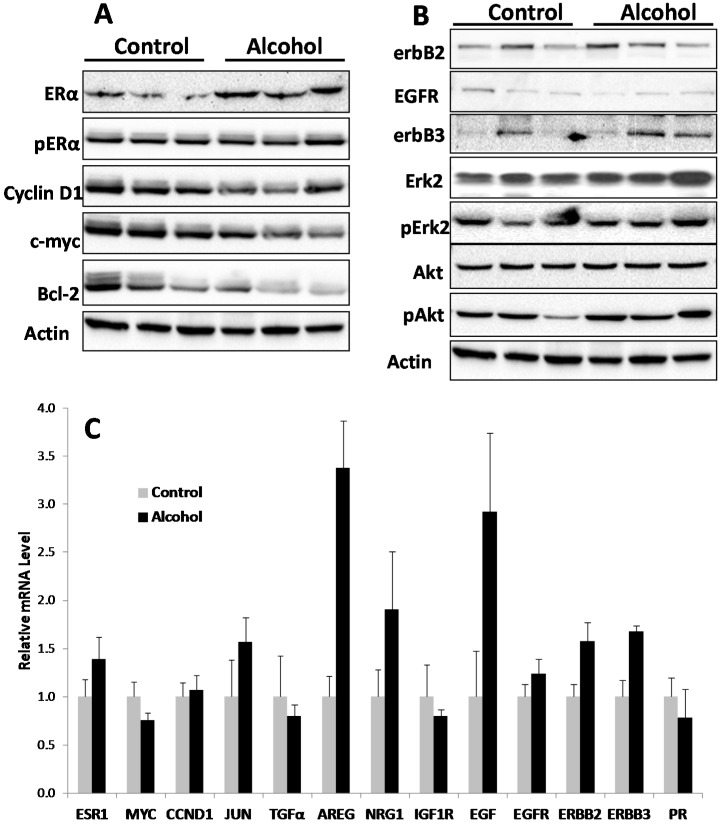
*In utero* exposure to alcohol induces the activation of the ER pathway at 10 weeks of age. Mammary tissues from control and alcohol-exposed mice were harvested at 10 weeks of age. Protein and mRNA levels of the indicated markers were detected using Western blot (**A**,**B**) and real time RT-PCR (**C**), respectively. Bars in C represent standard error of triplicate measurements in the qRT-PCR assay.

We next examined the effect of *in utero* exposure to low-dose alcohol on molecular changes in the ER and erbB-2 pathways in mammary tissues of 10-week-old mice. Although mammary whole mounts from the alcohol group displayed striking differences from control mammary whole mounts at this age, changes in the detected protein markers between the two groups were smaller than at five weeks. As shown in Figure 6A, among ER pathway markers, only protein levels of ERα were increased. For the erbB-2 pathway, the protein levels of pAkt, pErk, erbB-3 and erbB-2 were modestly increased (Figure 6B). At the transcriptional level (Figure 6C), the mRNA levels of the genes associated with the ER pathway were not increased. In contrast, the transcription of AREG, EGF, erbB-3 and erbB-2 was significantly increased in the alcohol group. The results suggest that, at 10 weeks of age, ER signaling induced by *in utero* alcohol exposure might have subsided and the modulation of the erB-2 pathway might be reflected in the selective upregulation of EGF-like ligands and downstream kinase activation.

### 2.7. In Utero Exposure to Low-Dose Alcohol Induces Increased Tumor Multiplicity and an Intriguing Mammary Tumor Development Pattern

**Figure 7:**
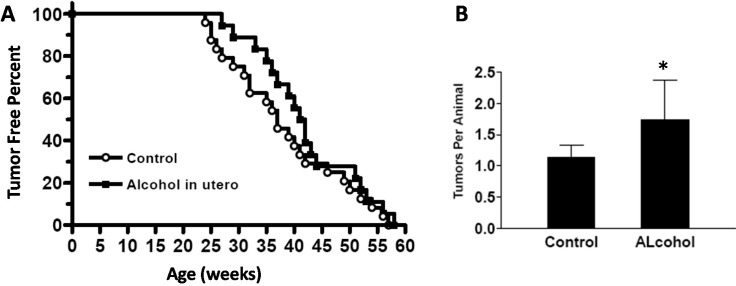
*In utero* exposure to alcohol modifies mammary tumor development in MMTV-erbB-2 transgenic mice. (**A**) Kaplan–Meier curves for tumor-free animals were calculated based on the tumor latency of the control (circle) and alcohol-exposed (square) mice. *p* > 0.05; and (**B**) Tumor multiplicity in MMTV-erbB-2 mice after different *in utero* treatments. Tumor multiplicity was based on the number of tumors in each animal by euthanization, when the first tumor reached 1.8 cm^3^ in volume. * *p* < 0.05.

Examination of mammary tumor development in MMTV-erbB-2 mice after *in utero* exposure to alcohol revealed an intriguing pattern (Figure 7). Although all mice in both groups developed tumors at approximately 58 weeks of age and there was no significant difference in tumor latency between the two groups (*p* = 0.3125), tumor development in the alcohol group showed a distinct pattern. In the control group, mice started to develop mammary tumors from 24 weeks of age, and the mean tumor latency was 37 weeks. In the alcohol group, tumor development started at 27 weeks, and the mean latency was 41 weeks; however, the rate increased after 40 weeks of age, suggesting that tumor development was promoted at older ages. Despite the insignificant difference in tumor latency, the tumor multiplicity (number of tumors per animal at the end point) of the alcohol group was greater than that of the control group (*p* = 0.039). Of note, mice in the alcohol group that had multiple tumors were the animals that developed tumors after 40 weeks of age, which suggests that *in utero* exposure to low-dose alcohol promotes tumor development at a later age. Preliminary examination showed that the majority of tumors from both groups were of the nodular, low grade, intermediate cell type histology, as reported in a previous study [23]. No significant differences in histopathological patterns between the two groups were observed (data not shown).

## 3. Discussion

The impact of *in utero* exposure to environmental or dietary factors on adult health and disease is an emerging field in biomedical research. The increased risk of breast cancer in later life induced by *in utero* alcohol exposure is of particular importance because of the common occurrence of alcohol drinking among pregnant women [10]. Previously, two studies showed that *in utero* alcohol exposure induced increases in tumor multiplicity in DMBA- or NMU-treated rats [12,13]. In one study, pregnant rats were fed liquid diets containing 16 g (7% alcohol of total energy) or 25 g (15% alcohol of total energy) alcohol/kg feed [12]. Based on a study on liquid diet consumption and alcohol intake [29], the alcohol doses used in this study would have been approximately 5 and 9 g/kg/day, respectively. In the other study, the dams were fed a liquid diet containing 6.7% ethanol from days 11 to 21 (35% of total calories) of gestation [13,14], which would have been a dose of approximately 16 g/kg/day. In the present study, pregnant MMTV-erbB-2 transgenic mice were exposed to 3–6 g/kg body weight of alcohol via the oral feeding of drinking water to mimic binge drinking. This is a much lower dose than in previous *in utero* exposure studies. Previous studies showed that mice administered 1.12 g/kg alcohol had in a BAC of 141 mg/dL at 30 min after administration [26]. In this study, the BAC of the pregnant erbB-2 mice 30 min after exposure to half of the daily dose was 114.5 mg/dL. Compared with previous reports, alcohol exposure in the pregnant mice in this study was in the low dose range.

Our results showed that *in utero* exposure to low-dose alcohol induced a series of alterations in mammary development and tumor risk in MMTV-erbB-2 transgenic mice. The notable changes included earlier onset of mammary development at puberty, longer extension of ductal trees at 10 weeks of age, and the upregulation of ER- and erbB-2-regulated signaling in the mammary tissues. *In utero* alcohol exposure induced an initial delay and later an acceleration of tumor development. Nevertheless, tumor multiplicity in these animals was increased. These results suggest that *in utero* exposure even to low doses of alcohol induces mammary reprogramming and has profound impacts on tumor risk later in life.

We found that *in utero* exposure to low doses of alcohol induced an intriguing tumor development pattern, which was an increase of tumor latency before 40 weeks of age. The rate of tumor formation was slow initially and then increased to catch up with the control group. The initial delay in tumor development was the opposite to what was expected. These data should be interpreted with caution. This delay might be partially explained by the weak proestrogenic effect induced by *in utero* exposure to low-dose alcohol. Previous studies showed that subtle changes in hormonal modulation may result in opposing effects on mammary tumor risk. For example, *in utero* exposure to genistein or n6 PUFA increased mammary tumor risk [5,30], whereas *in utero* exposure to soy protein isolate or n3 PUFA was associated with a protection from mammary tumor risk [6,7]. We also found that MMTV-erbB-2 mice implanted with low-dose estrogen pellets at a younger age had a slight delay in tumor latency but faster tumor development in the later stage, although implantation of high-dose estradiol (E2) pellets after puberty significantly promoted tumor development [23]. Opposite results from subtle changes of microenvironment were also observed in modified growth factor signaling. One of such examples is that inhibition of the IGF1 receptor promoted mammary tumor development in MMTV-Wnt mice [31]. In the present study, the weak estrogen-like effect associated with *in utero* exposure to a low dose of alcohol may induce altered differentiation that results in the initial delay in tumor development. Nevertheless, the modified tumor development pattern also includes relatively faster tumor development after 40 weeks and increased tumor multiplicity in mice that show a longer latency for tumor development, which could result from increased susceptibility to mutations due to altered hormonal and developmental conditions [32]. The specific mechanisms underlying these changes warrant further investigation. Based on the findings from this report, effects of *in utero* exposure to various doses of alcohol on mammary tumor risk in MMTV-erbB-2 transgenic mice will be followed.

We found that *in utero* exposure to alcohol induced significant changes in mammary morphogenesis in MMTV-erbB-2 mice. Previously, Hilakivi-Clarke *et al.* showed that *in utero* alcohol exposure induced increases in TEB numbers at puberty and mammary epithelial densities in 8- and 22-week-old rats [12]. Polano *et al.* reported that exposure to high-dose alcohol *in utero* had no effect on TEB numbers, although the proliferation index measured by BrdU incorporation was greater at PND 20 in the glands of the alcohol group [13,14]. In the present study, *in utero* alcohol exposure induced significant changes in mammary morphogenesis. Although the difference in TEB numbers in pubertal glands was not significant, mammary glands from mice that had undergone *in utero* alcohol exposure displayed prolonged extension of ductal trees at 10 weeks, a striking difference between the two groups. In particular, this change was accompanied by a reduction in the number of lateral buds and an increase in pubertal TEB-like structures at the distal end of the ductal tree compared to the controls. Our results suggest that *in utero* alcohol exposure not only induces ductal outgrowth but also alters mammary differentiation and the dynamics of morphogenesis, which results in the reprogramming of mammary development and tumor risk.

It has been reported that serum E2 levels were increased in pregnant rats exposed to alcohol and in offspring after *in utero* alcohol exposure, and these changes were associated with increased tumorigenesis [12,13,14]. Our data showed that increases in plasma E2 levels in alcohol-treated pregnant mice were not significant (Figure 1), which could be related to the lower dose of alcohol. Because *in utero* exposure to estrogen or estrogenic compounds has been associated with modified mammary tumor risk [30], the correlation between *in utero* exposure to alcohol-induced changes and altered E2 levels warrants further investigation. A dose-response relationship will be examined in future studies to establish this connection.

Cellular responses to these changes have not been extensively studied. In the present study, we focused on the regulation of the ER and erbB-2 pathways at both the protein and RNA levels in mammary tissues after different *in utero* treatments. Our results indicate that *in utero* exposure to low-dose alcohol induced significant upregulation of ERα expression and phosphorylation in mammary tissues at puberty. The molecular changes were more prominent in mammary tissues at puberty but less evident in 10-week-old animals. ER pathway activation in pubertal tissues was further supported by increased transcription/expression of classic ER target genes, including c-myc, cyclin D1, Bcl-2 and c-Jun, which are well established regulators of cell proliferation and survival. Activation of these potent molecules and the relevant pathways may partially explain the significant changes in mammary tissues. Interestingly, we noticed that although the ER pathway was generally activated, the mRNA levels of PR, a typical ER-responsive gene, were downregulated in mammary tissues after *in utero* alcohol exposure. This result differed from those of our studies on *in utero* exposure to genistein or bisphenol A (BPA), in which we observed increases in both ERα activation and PR mRNA levels in mammary tissues (unpublished data). The different effects of PR mRNA levels in this study might be related to a weaker estrogenic effect induced by *in utero* exposure to low-dose alcohol and the ability of this exposure to also induce changes in other PR regulators, which will be examined in future studies.

We examined molecular markers of the erbB family not just because erbB-2 is a predisposition factor of this transgenic model but also because regulation of the erbB family members plays a critical role in physiological mammary development and breast cancer pathogenesis [19,20]. We found that *in utero* exposure to low-dose alcohol induced modest phosphorylation/activation of Akt and Erk but had mixed effects on erbB receptors and ligands. Our results showed that *in utero* exposure to alcohol induced upregulation of erbB-3, Areg, NRG1 and TGFα transcription but downregulation of EGFR, erbB-2 and EGF at puberty. Among these proteins, Areg, NRG1, EGF and TGFα are the ligands for EGFR and/or erbB-3 [33]. In the mammary tissues of 10-week-old mice, mRNA levels of Areg and EGF were significantly upregulated in the alcohol group. As erbB-2 is an orphan receptor and its activation is mainly regulated by its interaction with activated family members upon ligand binding, modulation of the expression of these ligands by *in utero* exposure to alcohol would contribute to the modified signaling in the erbB-2 pathway and relevant phenotypic changes. For example, Areg and EGF specifically interact with the EGF receptor to promote the growth of normal epithelial cells [33], which may partially explain the prolonged ductal tree extension. Taken together, the data suggest that regulation of erbB family signaling is a critical target of cellular changes induced by *in utero* alcohol exposure. The underlying mechanisms of this effect and the impact of specific markers require further investigation. Moreover, to interpret the altered protein and mRNA markers, it is also possible that the shifts in signaling in the alcohol-treated animals reflect changes in the ratio of epithelial cells to stroma. This hypothesis will be tested by analyzing the relative composition of different mammary cell subtypes in future studies.

## 4. Experimental Section

### 4.1. Animals and Treatments

FVB/N-Tg/MMTV-erbB2 (MMTV-erbB-2) transgenic mice were purchased from the Jackson Laboratory (Bar Harbor, ME, USA). All animal experiments were approved by the Institutional Animal Care and Use Committees (IACUC) of North Carolina Research Campus (NCRC). The animals were fed a phytoestrogen-free AIN-93G semipurified diet (Bio-Serv Co., Frenchtown, NJ, USA) *ad libitum*. To prepare pregnant mice, MMTV-erbB-2 breeding pairs were mated at 8 weeks of age. For alcohol treatment, pregnant mice were exposed to alcohol via gavage feeding of alcohol water (diluted from 200 proof, undenatured ethanol) twice a day between days 10 and 19 of gestation. The doses for each feeding were: 0.5 mL of 10% alcohol on gestation days 10–12; 0.5 mL 20% alcohol on gestation days 13–15; 0.6 mL 20% alcohol on gestation days 16–19. The estimated alcohol dosages were 3 g/kg/day between day 10 and day 12 and 6 g/kg/day between day 13 and day 19 of gestation. In parallel, pregnant mice in the control group were fed with water. The body weights at the end of gestation for control and alcohol treated mice were 32.95 ± 1.24 and 32.93 ± 1.54 g, respectively. We did not see weight gain among alcohol-fed dams, possibly owing to changes in food intake patterns associated with acute alcohol exposure. For each group, 15 pregnant dams were treated. Five from each group were used for blood collection by cardiac puncture on day 18 of gestation. The offspring from the rest of the dams were left with mothers to nurse. They were pooled after weaning and then randomly selected for each parameter.

To study the effect of *in utero* alcohol exposure on mammary development and tumor risk, female mice that had undergone *in utero* treatments were examined as follows. The newborns in each group were weighed each week for 4 weeks. Vaginal opening of the offspring was also examined daily starting from PND 20 to determine the vaginal opening dates. At 5 and 10 weeks of age, 10 offspring mice from each group at each point were euthanized for whole mount, protein and RNA analyses. An additional 20 offspring mice from each group were used for tumor development analysis. To this end, the mice were monitored twice a week, and the dates of tumor appearance and tumor volume were recorded. Per IACUC protocol, the animals were euthanized when tumor volume reached 1.8 cm^3^. The tumor latency was defined as the time to first tumor appearance by palpation. Tumor multiplicity was based on the number of tumors in each animal before euthanization. Kaplan–Meier curves were used to document tumor-free survival.

### 4.2. Blood Alcohol Concentration (BAC) Determination

To measure BAC, pregnant mice from the alcohol and control groups were exposed to alcohol or water as scheduled above. On Day 18 of gestation, blood was collected by tail clipping 30 min after a dose of 500 µL of 20% (*v*/*v*) ethanol solution or water via gavage. BAC was measured using an ethanol assay kit from Abcam (Cambridge, MA, USA) according to the manufacturer’s instructions. Briefly, blood samples from individual animals were diluted 1:10 in the assay buffer. The samples and the standard ethanol solutions were incubated with reaction mix containing an ethanol probe at 37 °C for 30 min in a 96-well plate. The plate was then read at 540 nm with a SynergyMx microplate reader. The BACs were calculated based on the standard curve and the dilution factor. The tests were based on 5 replicates from different animals.

### 4.3. Blood Estradiol Assay

Whole blood was collected by cardiac puncture on Day 18 in pregnant mice with *in utero* alcohol exposure as detailed above. Plasma was prepared by centrifugation at 3000 rpm at 4 °C for 20 min. Estradiol levels were measured using the estradiol enzyme immunoassay (EIA) kit from Cayman Chemical (Ann Arbor, MI, USA). The diluted sample and the standards AChE tracer and EIA antiserum were added to appropriate wells of a microtiter plate that was coated with anti-rabbit IgG. Following incubation at room temperature for 60 min, the wells were washed five times and developed with Ellman’s reagent for 60 min. Absorbance at 405 nm was measured using a SynergyMx microplate reader. Data based on 5 replicates from different animals are expressed as pg of estradiol per mL.

### 4.4. Whole Mount Analysis

Once the mice in specific groups reached the indicated end point, their inguinal (#4) mammary glands were collected and fixed with Carnoy’s fixative overnight, followed by re-hydration and carmine aluminum staining [23]. The glands were then dehydrated and cleared with xylene. Prepared whole mounts were documented via digital photography for morphology analysis. For mammary glands around pubertal stages, the number of terminal end buds (TEBs) in each gland was counted. For whole mounts from older animals, mammary gland morphology was characterized by ductal extension beyond the lymph node and ductal density, as indicated by lateral bud numbers and branching.

### 4.5. Western Blot Analysis

Mammary tissues from the 4th and 5th pair of glands were collected, followed by snap freezing in a liquid nitrogen tank. Thawed tissues were homogenized at 4 °C in lysis buffer to extract total protein. The protein was quantified using a bicinchoninic acid (BCA) kit. Protein extracts were electrophoresed on a 10%–12% SDS-PAGE and transferred to nitrocellulose paper. The blots were incubated with TBS containing 0.1% Tween 20% and 5% powdered milk for 1 h and then incubated overnight with primary antibodies. Antibodies against ERα, c-myc, ERK2, Cyclin D1, Bcl-2, and β-actin were from Santa Cruz Biotechnology (Santa Cruz, CA, USA); antibodies against p-ERα, erbB-2, EGFR, erbB-3, p-Erk, p-Akt and Akt were from Cell Signaling Technology (Beverly, MA, USA). The membranes were washed and incubated with HRP-labeled anti-mouse or anti-rabbit secondary antibodies (Thermo Scientific, Waltham, MA, USA) for 1 h. Signals were visualized using Amersham enhanced chemiluminescence (ECL) reagents (GE Healthcare, Barrington, IL, USA) and captured with a FluorChem E imager from ProteinSimple (San Jose, CA, USA).

### 4.6. Quantitative Real Time PCR

Total RNA was isolated from the third pair of mammary glands using a Qiagen RNeasy mini kit with on-column DNAse digestion (Qiagen, Mississauga, ON, USA) per the manufacturer’s instructions. Equal amounts of RNA from each of 4 mice from the same group were pooled before cDNA was reverse transcribed using an iScript Reverse Transcriptase kit (Bio-Rad, Hercules, CA, USA). Real time PCR was performed with murine gene-specific primers using a Bio-Rad CFX96 Real Time PCR System (Bio-Rad). PCR amplification was carried out in a 20-µL reaction volume containing 50 ng of cDNA, 2 µM each of forward and reverse primers, and 10 µL Fast SYBR Green Master Mix (Bio-Rad). The PCR reactions were initiated with denaturation at 95 °C for 5 min, followed by 40 amplification cycles at 95 °C for 15 s and 60 °C for 1 min. Samples were run in triplicate, and data were normalized to beta actin before statistical analysis. The primers used in this study are listed in Table 2.

**Table 2:** List of primers used for qRT-PCR.

Genes	Forward	Reverse
*ACTB*	TTGCCGACAGGATGCAGAAGGA	AGGTGGACAGCGAGGCCAGGAT
*AREG*	ACCTGGAGGTGGTGACATGCA	TGCCGATGCCAATAGCTGCGA
*CCND1*	GGGTGGGTTGGAAATGAAC	TCCTCTCCAAAATGCCAGAG
*EGF*	TTTTGCCTCAGAAGGAGTGG	GGCCACACTTGGCAGTATATC
*EGFR*	ATCCTCTGCAGGCTCAGAAA	GGCGTTGGAGGAAAAGAAAG
*ERBB2*	GTCGCAACTTCATGTCGGTA	GATCATCATGGAGCTGGC
*ERBB3*	GACCTTCCAGACTCCGTTTG	AAATGGCCTGCAGCTTACAC
*IGF1R*	GGCAGCACTCGTTGTTCTC	GTACAACTACCGCTGCTGGA
*IGF2R*	TGCACACTCTTCTTCTCCTGGCA	GCAGATGTTGATATAGAAGTCAGG
*JUN*	GGGACACAGCTTTCACCCTA	GAAAAGTAGCCCCCAACCTC
*MYC*	TGAAGTTCACGTTGAGGGG	AGAGCTCCTCGAGCTGTTTG
*NRG1*	TTCATCACAACCCTGCACAT	GAACTTGGGTTGCTGTCCAT
*PR*	CACAGCGCTTCTACCAACTCACAA	TTGGGCAACTGGGCAGCAATAA

### 4.7. Statistical Analysis

Kaplan–Meier analyses and the log-rank test were used for tumor-free survival analysis. Dates of vaginal opening were analyzed using non-parametric test. Student’s *t*-test was used for the other tests.

## 5. Conclusions

Taken together, the data presented in this study demonstrated that *in utero* exposure to low-dose alcohol in MMTV-erbB-2 transgenic mice induced reprogramming of mammary development that resulted in significant changes in mammary morphogenesis, signaling in the ER and erbB-2 pathways, tumor multiplicity and development patterns. However, the results from this transgenic mouse model-based pilot study also raise many questions, including how the treatment induced the intriguing tumor latency patterns and how the modified gene expression profile relates to these phenotypic changes. Nevertheless, the results in this report have established a basis for further systematic investigation of breast cancer risk associated with *in utero* alcohol exposure. Whether and how the modulation of epigenetic regulation and the reprogramming of mammary stem cells are involved in this process will be interesting topics to explore. 
